# Gestational age-dependency of height and body mass index trajectories during the first 3 years in Japanese small-for-gestational age children

**DOI:** 10.1038/srep38659

**Published:** 2016-12-09

**Authors:** Kaori Maeyama, Ichiro Morioka, Sota Iwatani, Sachiyo Fukushima, Daisuke Kurokawa, Keiji Yamana, Kosuke Nishida, Shohei Ohyama, Kazumichi Fujioka, Hiroyuki Awano, Mariko Taniguchi-Ikeda, Kandai Nozu, Hiroaki Nagase, Noriyuki Nishimura, Chika Shirai, Kazumoto Iijima

**Affiliations:** 1 Department of Pediatrics, Kobe University Graduate School of Medicine, Kobe 6500017, Japan; 2 Kobe City Public Health Center, Kobe, Japan

## Abstract

Gestational age (GA) is thought to affect height growth in small-for-gestational age (SGA) children. However, the GA-specific trajectories in body mass index (BMI) and early appearances of adiposity rebound (AR) have not been fully investigated in a cohort of Japanese SGA children. A longitudinal cohort study was conducted with 1063 SGA children born in Kobe, Japan, with sufficient records from birth to 3 years of age. Subjects were divided into subgroups based on GA: 39–41 weeks GA (n = 723), 37–38 weeks GA (n = 256), 34–36 weeks GA (n = 62), and <34 weeks GA (n = 22). Height and BMI were assessed at 4 months, 9 months, 1.5 years, and 3 years of age. The catch-up rate for height was GA-dependent. Most children with 39–41 weeks GA (91%) caught up by 4 months of age; however, lower GA was associated with a slower elevation in the catch-up rate. The BMI trajectory during the first 3 years was also GA-dependent, with a change in GA dependency at a boundary of 37 weeks GA. Approximately 7% of SGA children had already developed AR before 3 years of age. In conclusion, growth patterns during infancy and early childhood in SGA children differ depending on GA.

Small-for-gestational age (SGA) children, who have growth restriction *in utero* resulting mainly in low birth weight, are at high-risk for growth impairment, including short stature in child- and adulthood^1^,^2^,^3^,^4^. Children born with low birth weight or SGA have been associated with increased morbidity of obesity and non-communicable diseases, such as coronary heart disease and type 2 diabetes (Developmental Origins of Health and Disease, DOHaD)^5^,^6^,^7^, suggesting that low birth weight or SGA infants suffer lifelong health disadvantages. It is important to know postnatal growth trajectories in Japanese SGA children, because the growth trajectories observed in this study can compare with “optimal” trajectories described for other populations^8^.

An early appearance of adiposity rebound (AR), the age at which the body mass index (BMI) starts to increase after infancy^9^,^10^,^11^,^12^, has been reported to be related to the development of obesity and type 2 diabetes, and may be a predictive marker^7^,^9^,^10^,^11^,^13^. The adiposity pattern of SGA children early in life has attracted worldwide attention in recent years. The number of longitudinal prospective cohort studies examining the BMI trajectory of SGA children early in life is limited, especially in Japan^14^. Previous reports have shown that gestational age (GA) might affect the height growth in SGA children^3^,^15^,^16^. GA-dependent changes in BMI and the early appearance of AR has not been fully studied in SGA children during infancy and early childhood. The aim of the current study was to examine the potential GA-dependency of not only height, but also the BMI trajectory during the first 3 years of life in SGA children using a Kobe-city cohort in Japan. We also investigated whether SGA children are at risk for an early appearance of AR depending on GA.

## Results

### The prevalence and characteristics of SGA children

**Figure 1:**
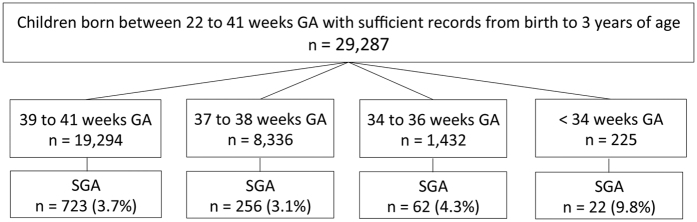
Flow chart for subject and classification. GA, gestational age; SGA, small-for-gestational age.

Of the 29,287 children born in Kobe, Japan, with sufficient records from birth to 3 years of age, 1063 (3.6%) were SGA. The prevalence of SGA within each GA subgroup is presented in Fig. 1. The prevalence of SGA for <34 weeks GA (but not 34–36 weeks or 37–38 weeks GA) was significantly higher compared to that for 39–41 weeks GA (p < 0.001). The background characteristics at birth are shown in Table 1. As expected, birth weight (BW) and birth length (BL) decreased with decreasing GA. The proportion of males in the 39–41 weeks GA subgroup was significantly lower than that in the 34–36 weeks GA subgroup (p < 0.001). The proportion of first births in the 39–41 weeks GA subgroup was significantly higher than that in the 37–38 weeks (p < 0.01) and <34 weeks GA subgroups (p < 0.05). No significant differences were found in the rate of maternal smoking.

### Trajectory of the standard deviation score and catch-up rate for height

**Figure 2:**
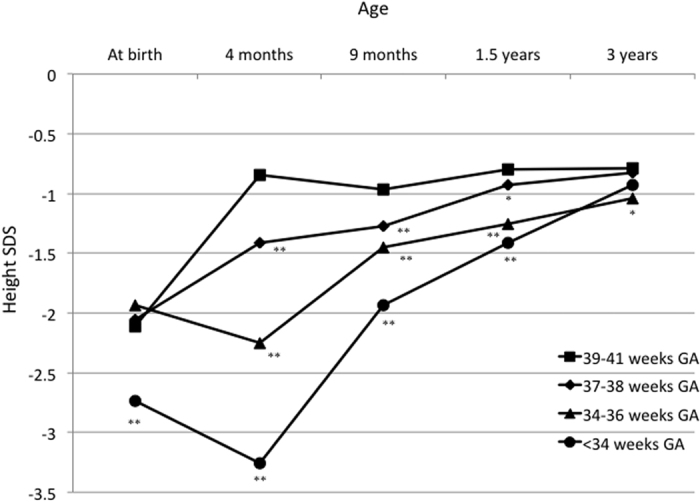
Gestational age-specific trajectories for height standard deviation scores. GA, gestational age; SDS, standard deviation score. ^*^p < 0.05 and ^**^p < 0.01 compared with 39–41 weeks GA.

The GA-specific trajectories for height standard deviation score (SDS) are shown in Fig. 2 and Supplementary Table S1. The mean height SDSs for SGA children with 39–41 and 37–38 weeks GA increased with increasing age. In contrast, the mean height SDSs for SGA children with 34–36 and <34 weeks GA decreased at 4 months of age, and increased thereafter. The mean height SDSs were significantly lower in the 37–38, 34–36, and <34 weeks GA subgroups compared with that in 39–41 weeks GA subgroup from 4 months to 1.5 years of age. At 3 years of age, the 37–38 and <34 weeks GA subgroups were no longer significantly different from the 39–41 weeks GA subgroup; however, the mean height SDS was still significantly lower in the 34–36 weeks GA subgroup (p = 0.044).

**Figure 3:**
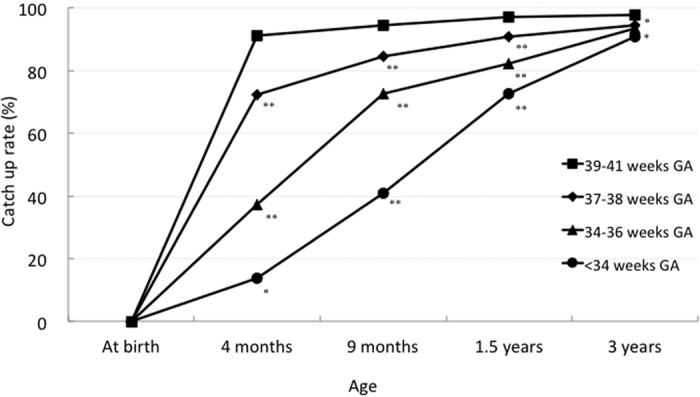
Gestational age-specific trajectories for the catch-up rate for height. At birth, the catch-up rate for small-for-gestational age children was defined as 0%. GA, gestational age. ^*^p < 0.05 and ^**^p < 0.01 compared with 39–41 weeks GA.

GA-specific trajectories of the catch-up rate for height are shown in Fig. 3 and Supplementary Table S1. The majority of SGA children with 39–41 weeks GA (91.0%) caught up at 4 months of age; however, for SGA children with lower GA, the catch-up rate had a slower elevation. The catch-up rates were significantly lower in the 37–38, 34–36, and <34 weeks GA subgroups compared with that in the 39–41 weeks GA subgroup from 4 months to 1.5 years of age. At 3 years of age, the 34–36 weeks GA subgroup was no longer significantly different from the 39–41 weeks GA subgroup (p = 0.054); however, the catch-up rates in the 37–38 and <34 weeks GA subgroups were still significantly different compared with that in the 39–41 weeks GA subgroup (p = 0.014; p = 0.048, respectively).

### Trajectory of BMI SDS

**Figure 4:**
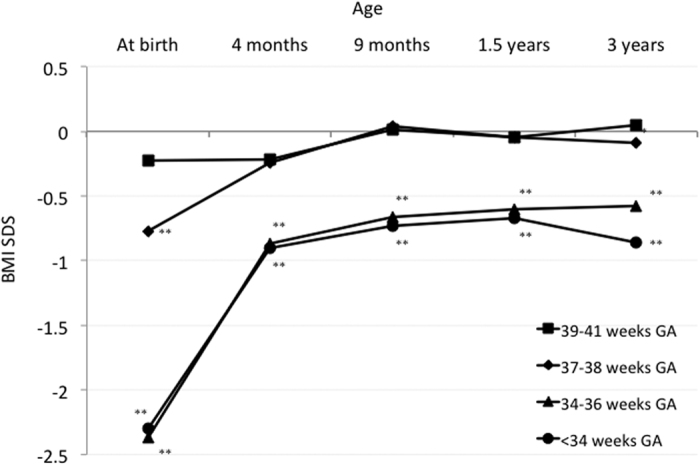
Gestational age-specific trajectories for body mass index standard deviation scores. BMI, body mass index; GA, gestational age; SDS, standard deviation score. ^**^p < 0.01 compared with 39–41 weeks GA.

GA-specific trajectories of BMI SDS are shown in Fig. 4 and Supplementary Table S1. The trajectories of mean BMI SDS during the first 3 years of life for the 39–41 and 37–38 weeks GA subgroups were clearly separated from the 34–36 and <34 weeks GA subgroups. The mean BMI SDSs for SGA children with 39–41 and 37–38 weeks GA were around −0.2 SDS at 4 months of age and stayed around 0 SDS thereafter. In contrast, the mean BMI SDSs for SGA children with 34–36 and <34 weeks GA increased from around −2.3 SDS at birth to −0.9 SDS at 4 months of age, then stayed between −1.0 and −0.5 SDS thereafter. The mean BMI SDSs were significantly lower in the 34–36 weeks and <34 weeks GA subgroups compared with that in the 39–41 weeks GA subgroup from 4 months to 3 years of age. In contrast, the mean BMI SDSs did not differ between the 37–38 weeks and 39–41 weeks GA subgroups.

### SGA children with AR

Table 2 shows the mean ⊿BMI in SGA children for each GA subgroup as well as the proportion of children with AR (increased BMI between at 1.5 years of age and at 3 years of age). The mean ⊿BMI was below 0 (mean ⊿BMI, −0.35); however, 7.1% of SGA children had AR, indicating that some had a ⊿BMI of more than 1.0. AR did not occur at all in SGA children with <34 weeks GA, but this was not a statistically significant finding because of the small number in <34 weeks GA subgroup (n = 22) and the low frequency of the event (7%) in all subjects.

## Discussion

Using a Japanese cohort, our longitudinal study of SGA children revealed that the height trajectory during the first 3 years of life is GA-dependent. Although the proportion of males in the 34–36 weeks GA subgroup was high probably due to a small number (n = 62), no significant differences were observed in growth trajectories between male and female children in that subgroup (data not shown). As age increases, the rate of height catch-up occurs in the following order (fastest to slowest): 39–41, 37–38, 34–36, and <34 weeks GA. Although the BMI trajectory during the first 3 years is also GA-dependent, the pattern was different with a change in GA dependency occurring at a boundary of 37 weeks GA. We also found that AR occurred from 1.5 to 3 years of age in SGA children with ≥34 weeks GA, but not in those with <34 weeks GA. These results suggest GA should be considered when following the growth of SGA children during infancy and early childhood.

Previous studies have reported the catch-up rate for height in SGA children during early childhood. For example, Albertsson-Wikland *et al*. reported that 87% of SGA children (defined as below −2.0 SDS in birth weight and length based on GA) with ≥37 weeks GA showed catch-up growth within 2 years of age^17^. In addition, 87.5% and 82.5% of SGA children (defined as below the 3rd percentile in birth weight and length based on GA) with ≥37 weeks and <37 weeks GA, respectively, have been reported to show catch-up growth during the first 2 years of life, suggesting that while SGA children with <37 weeks GA have a lower catch-up rate at 2 years of age than those with ≥37 weeks GA, it is without significant difference^15^. However, in a Japanese cohort, Itabashi *et al*. reported that 92%, 91%, and 74% of SGA children with ≥37 weeks GA, 32–36 weeks GA, and <32 weeks GA, respectively, caught-up in height at 3 years of age, with a significantly lower catch-up rate in SGA children with <32 weeks GA compared with that in the other GA groups^3^. This result was similar to the results of the current study. SGA children with <34 weeks GA demonstrated a lower catch-up rate at 3 years of age and may be at a higher risk of short stature compared with SGA children with ≥37 weeks GA.

Similar to previous reports^3^,^15^, the current study demonstrated that the rate of catch-up depended on GA. While 91% of SGA children with ≥39 weeks GA had caught up by 4 months of age, only 14% of SGA children with <34 weeks GA were caught up by this age. At 3 years of age, the catch-up rate was 98% and 91% in SGA children with ≥39 weeks GA and <34 weeks GA, respectively. Therefore, the possibility of catch-up in height by 3 years of age is likely to be low when SGA children with ≥39 weeks GA have not caught-up by 4 months of age, while SGA children with <34 weeks GA are expected to take a long time to catch-up and year-based time would be needed to catch-up. SGA children with <34 weeks GA should be closely followed in terms of height for at least a couple of years and growth hormone therapy should be considered if they do not catch up in height^18^,^19^.

The current study revealed for the first time in a Japanese population that during the first 3 years of age, SGA children with <37 weeks GA had significantly lower BMI SDS compared with SGA children with ≥37 weeks GA. Taal *et al*. reported that the BMI in SGA children without catch-up was low at 4 years of age^20^. The BMI in very low birth weight children has been shown to be significantly lower than that in normal birth weight children at 8 years of age^21^. A greater percentage of underweight and a reduced percentage of overweight adult individuals has reported for SGA children with <37 weeks GA compared with children with ≥37 weeks GA, with only ~5% SGA children with <37 weeks GA developing obesity^22^. Despite differences in the observed age groups, these reports suggest that SGA children, especially those with a preterm birth (<37 weeks GA), may not develop obesity thereafter. On the other hand, Gaskins *et al*. reported that SGA children with <33 weeks GA are at risk for being overweight at 11 years of age^23^. Thus, although SGA children with <37 weeks GA generally appear to have low BMI during infancy and early childhood, some of them may be an at-risk population for obesity in later childhood and adulthood. Further studies are needed to confirm this speculation.

As the establishment of the DOHaD, low birth weight or SGA children have been shown to be at risk for obesity and non-communicable diseases^5^,^6^,^7^. The early appearance of AR is reported to be a predictive marker of obesity, non-communicable diseases, and metabolic syndrome^7^,^9^,^10^,^11^,^13^. Therefore, we investigated whether the early appearance of AR occurred in SGA children. Previous reports similarly defined early AR as the appearance of increased BMI at ≤4 years of age^7^,^13^. Although the mean ⊿BMI was below 0, 7.1% of SGA children had already developed AR before 3 years of age. In addition, all children with an early appearance of AR were born with ≥34 weeks GA. Meas *et al*. reported that the proportion of obesity was increased at 30 years of age for SGA individuals with ≥37 weeks GA^24^. Similarly, Gaskins *et al*. reported that not only SGA, but also high BW were associated with being overweight or obese at 11 years of age for children with 32–36 weeks GA^23^. Based on these results, we speculate that SGA children born ≥37 weeks GA or 34–36 weeks GA are at potential risk for the development of early AR, which may lead to the development of obesity, non-communicable diseases, and metabolic syndrome during late childhood and adulthood.

Some limitations exist in the current study. (1) The cohort of SGA children with 34–36 and <34 weeks GA was relatively small compared with the size of the other GA groups, therefore, the results in SGA children with 34–36 and <34 weeks GA cannot draw the conclusion definitely. (2) A growth is affected by the nutrients and cares, however, we could not confirm whether all SGA children received the same nutrients and cares according to a recommendation for Japanese^25^. (3) Obstetric causes resulted in preterm birth could not be obtained in preterm SGA children. (4) The data of parents’ height and weight to predict the physique for their children also could not be obtained. Finally, (5) although we followed SGA children for 3 years of age, a longer follow-up period would be ideal in order to assess the association between AR and obesity/metabolic syndrome in school-aged children. Despite these limitations, this is the first report showing a difference in growth patterns for 3 years of age based on GA using a homogeneous Japanese cohort.

In conclusion, the BMI trajectory during the first 3 years for SGA children is GA-dependent. Furthermore, some SGA children show an early appearance of AR and may be at increased risk for the development of obesity, non-communicable diseases, and metabolic syndrome later in life. Therefore, GA should be considered in long-term studies of the health risks associated with SGA.

## Methods

### Study design

A longitudinal cohort study of 29,287 children born between January 2006 and December 2009 in Kobe, Japan was conducted. Written informed consent was obtained from the parents. The following anonymous data collected at birth were extracted from maternity health records: GA, BW, BL, sex, the mother’s history of childbirth, and smoking status during pregnancy. The BW and BL were measured at each hospital or maternity clinic where the children were born. Thereafter, a regular physical examination was performed at the Kobe City Public Health Center or pediatric outpatient clinics at around 4 months, 9 months, 1.5 years, and 3 years of age (uncorrected). The practical median age (range) of the physical examinations was 4 (3–8), 9 (8–12), 19 (17–23), and 39 (38–47) months of age.

Records regarding body weight and height were collected at the Kobe City Public Health Center. After approval by the review board at the Planning and Coordination Bureau of the city of Kobe (approved on the 1^st^ of April, 2015), analyses were carried out at the Kobe University Graduate School of Medicine. The study methods were carried out in accordance with approved guidelines.

### Subjects

All of 29,287 children with a GA between 22 to 41 weeks and sufficient records from birth to 3 years of age were included in the current study. Children born with a GA ≥42 weeks were excluded due to the lack of Japanese standard data regarding BW and BL for this GA category. The final sample was classified into 4 subgroups based on GA at birth: 39–41 weeks GA (n = 19,294), 37–38 weeks GA (n = 8,336), 34–36 weeks GA (n = 1,432), and <34 weeks GA (n = 225) (Fig. 1).

### Physical measurements

BW for each age point, including at birth, was measured using a scale. BL/height at birth, 4 months, 9 months, and 1.5 years of age was defined as the crown-to-heel length determined using a measuring tape or ruler by midwives or trained nurses. The height at 3 years of age was measured to the nearest 0.1 cm using a stadiometer by public health nurses or trained nurses.

BMI was calculated as BW [kg] divided by (height [m])^2^. SDSs for BW, BL, height, and BMI at each age point were calculated using nordiFIT software^®^ (Novo Nordisk Pharma Ltd., Tokyo, Japan)^26^,^27^. This software can calculate SDS based on the sex-specific standards for BW and BL by GA, or height and BMI by age in a Japanese population^26^,^27^. SDSs for height and BMI were adjusted by the age- and sex-specific standards to compare at the different age points.

For the height and BMI trajectories, the mean value and SDS of the height and BMI at each age point were calculated within GA subgroups and a line graph was constructed by connecting the values at the observed age points.

### SGA

SGA was defined as ≤−2.0 SDS for BW and/or BL based on Japanese neonatal anthropometric charts at birth for GA, sex, and the mother’s history of childbirth^26^. The prevalence of SGA was calculated for each GA subgroup.

### Catch-up

Catch-up was defined as reaching >−2.0 SDS in height. At birth, the catch-up rate for SGA children was defined as 0%. For each GA subgroup, the catch-up rate for height is shown at each age point as the percentage of SGA children achieving catch-up.

### Adiposity rebound

BMI generally peaks during the first year of life and subsequently declines, reaching a nadir at ~6 years of age^9^,^10^,^11^,^12^,^13^. Therefore, in the current study, AR was defined as a >1.0 rise in BMI between 1.5 to 3 years of age. For the analysis of AR, ⊿BMI was defined as [BMI at 3 years of age] - [BMI at 1.5 years of age] and the number (%) of SGA children with AR was examined for each GA subgroup.

### Statistical analysis

Data are presented as mean (±standard deviation), median (range), or number (percentage). The data for 37–38 weeks GA, 34–36 weeks GA, and <34 weeks GA were compared with those for 39–41 weeks GA, which served as a control. Chi-square tests (2 × 2 table analysis) and Student’s t-tests were performed using Excel Statistics (Statcel 3; Social Survey Research Information Co. Ltd., Tokyo, Japan). A p < 0.05 was considered statistically significant.

## Additional Information

**How to cite this article**: Maeyama, K. *et al*. Gestational age-dependency of height and body mass index trajectories during the first 3 years in Japanese small-for-gestational age children. *Sci. Rep.* **6**, 38659; doi: 10.1038/srep38659 (2016).

**Publisher's note:** Springer Nature remains neutral with regard to jurisdictional claims in published maps and institutional affiliations.

## Supplementary Material

Supplementary Table

## Figures and Tables

**Table 1:** Characteristics of small-for-gestational age children at birth.

	Term birth		Preterm birth	
	39–41 weeks GA n = 723	37–38 weeks GA n = 256	34–36 weeks GA n = 62	<34 weeks GA n = 22
GA	39.8 ± 0.75	37.6 ± 0.49**	35.3 ± 0.78**	31.4 ± 1.94**
BW, g	2554 ± 286	2216 ± 336**	1682 ± 265**	1189 ± 364**
BW SDS	−1.72 ± 0.87	−1.79 ± 1.00	−2.34 ± 0.69**	−2.19 ± 1.37
BL, cm	45.6 ± 1.49	43.5 ± 1.93**	41.0 ± 2.29**	34.6 ± 4.23**
BL SDS	−2.11 ± 0.72	−2.06 ± 0.75	−1.93 ± 0.69	−2.74 ± 1.07*
Male (%)	285 (39.4)	110 (43.0)	43 (69.4) **	8 (36.4)
First birth (%)	444 (61.4)	128 (50.0) **	33 (53.2)	8 (36.4) *
Maternal smoking (%)	76 (10.5)	28 (10.9)	3 (4.8)	2 (9.1)

Data are shown as mean ± standard deviation or number (%). BL, birth length; BW, birth weight; GA, gestational age. ^*^p < 0.05 and ^**^p < 0.01 compared with 39–41 weeks GA.

**Table 2:** ⊿Body mass index and adiposity rebound in small-for-gestational age children classified by gestational age.

	n	⊿BMI	With AR
39–41 weeks GA	723	−0.29 ± 0.96	54 (7.5)
37–38 weeks GA	256	−0.47 ± 0.89	17 (6.6)
34–36 weeks GA	62	−0.45 ± 0.76	4 (6.5)
<34 weeks GA	22	−0.58 ± 0.63	0 (0.0)
Total	1063	−0.35 ± 0.93	75 (7.1)

Data are shown as number (%) or mean ± standard deviation. ⊿BMI is [BMI at 3 years of age] - [BMI at 1.5 years of age]. AR, adiposity rebound; BMI, body mass index; GA, gestational age.
