# Supplementary material for: Gestational age-dependency of height and body mass index trajectories during the first 3 years in Japanese small-for-gestational age children
Source: Sci Rep. 2016 Dec 9;6:38659. doi: 10.1038/srep38659 (PMC5146673; doi:10.1038/srep38659)
Supplement: Supplementary Table [file srep38659-s1.pdf]

Gestational age-dependency of height and body mass index trajectory during the first 3 years in Japanese small-for-gestational age children

Kaori Maeyama[1], Ichiro Morioka[1,*], Sota Iwatani[1], Sachiyo Fukushima[1], Daisuke Kurokawa[1], Keiji Yamana[1], Kosuke Nishida[1], Shohei Ohyama[1], Kazumichi Fujioka[1], Hiroyuki Awano[1], Mariko Taniguchi-Ikeda[1], Kandai Nozu[1], Hiroaki Nagase[1], Noriyuki Nishimura[1], Chika Shirai[2], Kazumoto Iijima[1]

[1]Department of Pediatrics, Kobe University Graduate School of Medicine, Kobe 6500017, Japan

[2]Kobe City Public Health Center, Kobe 6508570, Japan

[*]Correspondence and requests for materials should be addressed to:

Ichiro Morioka, MD, PhD

Department of Pediatrics, Kobe University Graduate School of Medicine

7-5-2, Kusunoki-cho, Chuo-ku, Kobe 650-0017, Japan.

Phone: +81-78-382-6090, Fax: +81-78-382-6099, E-mail: ichim@med.kobe-u.ac.jp

Supplementary Table S1. Longitudinal data for height and body mass index in small-for-gestational age children

A. Height

	At birth	4 months	9 months	1.5 years	3 years
39–41 weeks GA	45.6 ± 1.49	61.1 ± 1.99	68.3 ± 2.31	78.2 ± 2.50	91.6 ± 3.25
37–38 weeks GA	43.5 ± 1.93	59.9 ± 2.23	67.7 ± 2.37	77.9 ± 2.75	91.5 ± 3.59
34–36 weeks GA	41.0 ± 2.29	58.6 ± 2.43	67.7 ± 2.63	77.4 ± 2.78	91.0 ± 2.75
<34 weeks GA	34.6 ± 4.23	56.9 ± 3.02	66.8 ± 2.88	76.6 ± 2.82	91.3 ± 3.49

B. Height SDS

	At birth	4 months	9 months	1.5 years	3 years
39–41 weeks GA	-2.11 ± 0.72	-0.84 ± 0.86	-0.97 ± 1.00	-0.80 ± 0.88	-0.79 ± 0.92
37–38 weeks GA	-2.06 ± 0.75	-1.41 ± 0.96**	-1.27 ± 0.94**	-0.93 ± 0.97*	-0.83 ± 1.01
34–36 weeks GA	-1.93 ± 0.69	-2.25 ± 1.01**	-1.45 ± 1.04**	-1.25 ± 0.93**	-1.04 ± 0.77*
<34 weeks GA	-2.74 ± 1.07**	-3.26 ± 1.28**	-1.93 ± 1.02**	-1.41 ± 1.02**	-0.93 ± 1.09

C. Catch-up rate for height

	At birth	4 months	9 months	1.5 years	3 years
39–41 weeks GA	0 (0.0)	658 (91.0)	682 (94.3)	701 (97.0)	706 (97.6)
37–38 weeks GA	0 (0.0)	185 (72.3)**	216 (84.4)**	232 (90.6)**	242 (94.5)*
34–36 weeks GA	0 (0.0)	23 (37.1)**	45 (72.6)**	51 (82.3)**	58 (93.5)
<34 weeks GA	0 (0.0)	3 (13.6)**	9 (40.9)**	16 (72.7)**	20 (90.9)*

D. BMI

	At birth	4 months	9 months	1.5 years	3 years
39–41 weeks GA	12.4 ± 1.74	16.7 ± 1.44	16.8 ± 1.41	15.8 ± 1.12	15.5 ± 1.14
37–38 weeks GA	11.8 ± 2.25	16.7 ± 1.37	16.9 ± 1.36	15.8 ± 1.31	15.3 ± 1.18
34–36 weeks GA	10.0 ± 1.60	15.9 ± 1.45	16.0 ± 1.39	15.2 ± 1.11	14.8 ± 1.18
<34 weeks GA	10.2 ± 4.21	15.8 ± 1.68	15.7 ± 1.92	15.0 ± 1.06	14.4 ± 1.06

E. BMI SDS

	At birth	4 months	9 months	1.5 years	3 years
39–41 weeks GA	-0.23 ± 1.53	-0.22 ± 0.99	0.014 ± 1.03	-0.052 ± 0.90	0.046 ± 0.89
37–38 weeks GA	-0.77 ± 1.98**	-0.24 ± 0.94	0.043 ± 0.98	-0.050 ± 1.04	-0.086 ± 0.97
34–36 weeks GA	-2.37 ± 1.47**	-0.87 ± 0.99**	-0.66 ± 1.02**	-0.60 ± 0.91**	-0.59 ± 1.02**
<34 weeks GA	-2.30 ± 3.71**	-0.90 ± 1.22**	-0.73 ± 1.44**	-0.67 ± 0.92**	-0.86 ± 0.99**

Data are shown as mean ± standard deviation and number (%). BMI, body mass index; GA, gestational age; SDS, standard deviation score. *$p < 0.05$ and **$p < 0.01$ compared with 39–41 weeks GA.
